# Clinical outcomes of submassive pulmonary embolism thrombolysis—an Indian experience

**DOI:** 10.1186/s43044-020-00123-8

**Published:** 2020-12-14

**Authors:** Nadeem U. Rehman, Mohd Iqbal Dar, Manish Bansal, R. R. Kasliwal

**Affiliations:** 1 Medanta Heart Institute, Delhi, India; 2 Department of Cardiology, SKIMS Soura, Srinagar, Jammu and Kashmir 190011 India

**Keywords:** Thrombolysis in pulmonary embolism, Submassive pulmonary embolism, RV systolic dysfunction, Pulmonary artery pressures

## Abstract

**Background:**

Acute pulmonary thromboembolism is the most dangerous presentation of venous thromboembolic disease. The role of thrombolysis in massive pulmonary embolism has been studied extensively, but the same is not there for submassive pulmonary embolism. This study is aimed at evaluating the effects of thrombolysis in acute submassive pulmonary embolism. This was a prospective, case-control, observational study. Patients presenting with acute submassive pulmonary embolism were divided into thrombolysis group and control group depending on whether they received thrombolysis plus anticoagulation or anticoagulation only, respectively.

**Results:**

A total of 86 patients were included in the study. Forty-two patients were in the thrombolysis group, and 44 patients were in the control group. The mean ± SD age in the control and thrombolysis groups was 63.3 ± 14.7 and 56.4 ± 13.8 years, respectively. The two groups were well matched in sex distribution and associated comorbidities like COPD, active surgery, major trauma, and immobilization. On echocardiography, dilated RA/RV in pre-treatment vs. post-treatment was seen in 20 (45.5%) vs. 20 (45.5%) in the control group and 26 (61.9%) vs. 11 (26.2%) in the thrombolysis group. Similarly, RV systolic dysfunction in pre-treatment vs. post-treatment was seen in 24 (54.5%) vs. 21 (47.7%) in the control group and 22 (52.4%) vs. 8 (19.0%) in the thrombolysis group. Pulmonary artery pressure in pre-treatment vs. post-treatment was 64.4 ± 15.0 vs. 45.9 ± 9.9 mmHg in the control group and 68.3 ± 17.4 vs. 31.4 ± 6.9 mmHg in the thrombolysis group. In control vs. thrombolysis group, there were 5 vs. 1 death, 6 vs. 1 hemodynamic decompensation, and 6 vs. 1 patient needing mechanical ventilation.

**Conclusion:**

Thrombolysis in submassive pulmonary embolism is associated with better right ventricular functions, lower pulmonary artery pressures, and comparable mortality rates.

## Background

The estimated incidence of venous thromboembolic disease (VTE) annually is about 1 to 2 persons per 1000 population. The manifestation of VTE can range from deep vein thrombosis (DVT) to pulmonary embolism (PE) or a combination of the two conditions [1–3]. Despite recent advances in treatment, a sizable portion of patients of acute pulmonary embolism and DVT who survive the initial disease are at risk of development of subsequent life-threatening complications like recurrent VTE, post-thrombotic syndrome, and chronic thromboembolic pulmonary hypertension (CTEPH) [1, 4]. The overall mortality rate of VTE in the first 3 months after diagnosis usually exceeds 15% [5].

Among the presentation of VTE, acute pulmonary embolism carries the highest mortality. Acute pulmonary embolism can present as massive, submassive, or non-massive or low-risk PE depending on the hemodynamic stability and right ventricular dysfunction as demonstrated by echocardiography, computed tomography, or cardiac biomarkers. The 90-day mortality in massive pulmonary embolism is 58.3% and 15.1% in submassive PE as per the International Cooperative Pulmonary Embolism Registry (ICOPER) [6]. Multiple other studies have demonstrated a mortality of 2% in patients presenting as low-risk pulmonary embolism [7–9]. Overall, 4% of patients who survive acute pulmonary embolism develop CTEPH subsequently [10].

Thrombolytic therapy for massive PE has been extensively evaluated and has been proven to reduce mortality with multiple guidelines (ACC, AHA, and ESC) advocating prompt thrombolysis in carefully chosen patients [11–13]. Thrombolytic therapy has been shown to reduce the incidence of CTEPH and improve quality of life. There are no clear guidelines for thrombolysis in submassive PE. Reduction in mortality by thrombolysis in submassive PE has been suggested by a recent meta-analysis [14]. This reduction in mortality was however found to be associated with increased morbidity due to increased incidence of major bleeding.

This study was aimed to evaluate the effects of thrombolysis and compare the clinical outcomes of thrombolysis plus standard treatment vs. standard treatment only in patients presenting as submassive pulmonary thromboembolism.

## Methods

A prospective, case-control study was conducted over a period of 2 years at this center. The ethical clearance for the study design was sought and granted by the institutional ethical committee (IEC). Patients were enrolled in this study from June 2017 to May 2019. Patients presenting to this center with submassive pulmonary embolism were enrolled in this study.

### Inclusion criteria

The inclusion criteria include an age of 18 years or older, objectively confirmed acute submassive pulmonary embolism (as defined subsequently) with an onset of symptoms 15 days or less before seeking medical consultation, right ventricular dysfunction confirmed by echocardiography or spiral computed tomography (CT) of the chest, and myocardial injury confirmed by a positive test for troponin I or troponin T.

### Exclusion criteria

Patients denying consent, having contraindication to thrombolysis, or having previous history of allergy to contrast dye were excluded from the study.

### Definitions [15]

*Massive pulmonary embolism* is defined as a conglomerate of pulselessness, persistent bradycardia with rate < 40 bpm, and signs of shock or sustained hypotension. Sustained hypotension includes systolic blood pressure (SBP) of < 90 mmHg for > 15 min, an SBP of < 100 mmHg in a patient with a history of hypertension, or a > 40% reduction in baseline SBP in the absence of dysrhythmia, hypovolemia, sepsis, or left ventricular (LV) dysfunction.

*Submassive embolism* is the one with normal or near-normal SBP (≥ 90 mmHg) with evidence of cardiopulmonary stress, including RV dysfunction or myocardial necrosis. RV dysfunction is defined by RV dilation on echo (RV diameter/LV diameter > 0.9), RV systolic dysfunction on echo, brain natriuretic peptide (BNP) elevation (> 90 pg/mL), N-terminal pro-BNP (> 500 pg/mL), or electrocardiographic (ECG) changes (new RBBB, anteroseptal ST ↑/↓, or anteroseptal T-wave ↓). Myocardial necrosis is defined as an elevation in troponin I or T over laboratory normal value or above patient baseline.

*Non-massive PE* is defined as no signs of clinical instability, hemodynamic compromise, and no RV strain on echocardiographic or elevation in biomarker.

The patient population was divided into two groups, the thrombolysis group and control group depending on whether the patient received thrombolysis plus anticoagulants or anticoagulants only by simple randomization method. The randomization was completed within 2 h of hospital admission. All the patients included in the study were concomitantly evaluated for the presence of underlying risk factors for PE development like COPD, congestive cardiac failure, previous PTE, active cancer, recent surgery or major trauma, pregnancy, immobilization, estrogen use, and DVT. Patients were also evaluated for the presence of inherited thrombogenic risk factors like factor V Leiden, prothrombin gene mutation, anti-thrombin III deficiency, protein C deficiency, protein S deficiency, ANA/APLA, JAK2, and homocysteinemia.

### Treatment

#### Thrombolysis arm

The patients in the thrombolysis arm received a single bolus dose of 100 mg of alteplase followed by a maintenance dose of unfractionated heparin to maintain APTT between 2 and 2.5 times the upper limit of the normal range.

#### Control arm

The patients in the control arm received an initial bolus dose of unfractionated heparin and subsequent maintenance dose. The heparin infusion rate was adjusted to achieve and maintain an APTT that was 2 to 2.5 times the upper limit of the normal range.

### Follow-up

All the patients were followed for 30 days and were evaluated for death, hemodynamic decompensation (or collapse), bleeding, stroke, recurrent pulmonary embolism, and serious adverse events. The primary outcome was the clinical composite of death from any cause, hemodynamic decompensation (or collapse), and need for mechanical ventilation within 7 days of hospitalization. The secondary outcomes included death within 7 days after hospitalization, hemodynamic decompensation within 7 days, confirmed symptomatic recurrence of pulmonary embolism within 7 days, death within 30 days, and major adverse events in 30 days. Safety outcomes were defined as ischemic or hemorrhagic stroke (including hemorrhagic conversion of ischemic stroke) within 7 days after hospitalization, extracranial major (moderate or severe) bleeding within 7 days, and serious adverse events within 30 days.

### Statistical analysis

The analysis included profiling of patients on different demographic, clinical, and radiological findings. Quantitative data were presented in terms of means and standard deviation. Qualitative/categorical data were presented as absolute numbers and proportions. Student’s *t* test was used for the comparison of quantitative outcome parameters for normally distributed data, and Wilcoxon’s test was used for non-normally distributed data. For paired comparison, Student’s *t* test and sign rank test were used for normally and non-normally distributed data, respectively. *p* value <0.05 is considered statistically significant. SPSS software version 20.0 was used for statistical analysis.

## Results

**Fig. 1:**
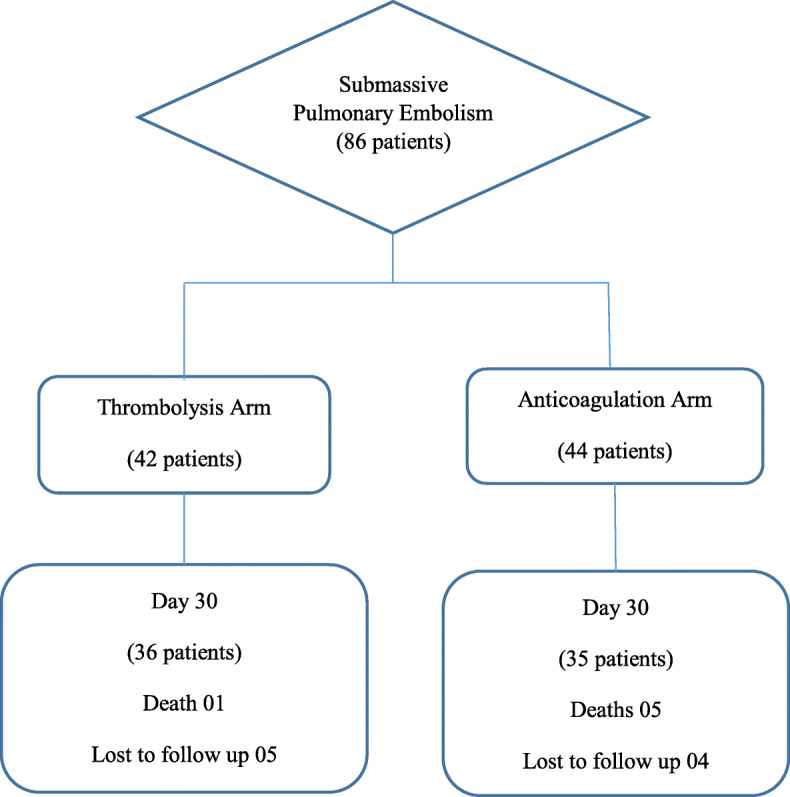
Overview of the study

A total of 86 patients were included in the study. Forty-two patients were in the thrombolysis arm, and 44 were in the anticoagulation arm. The overview of the study is given in Fig. 1.

The demographic features of the study population are given in Table 1.

**Table 1:** Demographic features of the study population

		Control group, *n* = 44	Thrombolysis group, *n* = 42	*p* value*
Sex	Male	24 (54.5)	29 (69)	0.167
	Female	20 (45.5)	13 (31)	
Age, mean ± SD (range)		63.3 ± 14.7 (28–86)	56.4 ± 13.8 (20–69)	0.001
Weight, mean ± SD (range)		73.5 ± 11.6 (40–96)	55 ± 16.5 (16–86)	0.611
Comorbidities	COPD	4 (9.1)	3 (7.1)	0.741
	CCF (RHF)	5 (11.4)	12 (28.6)	0.045
	Previous PTE	0 (0)	1 (2.4)	0.303
	Active cancer	4 (9.1)	3 (7.1)	0.741
	Surgery and major trauma	1 (2.3)	6 (14.3)	0.042
	Pregnancy	0 (0)	1 (2.4)	0.303
	Immobilization	2 (4.5)	4 (9.5)	0.365
	Estrogen use	1 (2.3)	2 (4.8)	0.529
	DVT	5 (11.4)	4 (9.5)	0.781

*p value < 0.05, statistically significant. *COPD* chronic obstructive pulmonary disease, *CCF* congestive cardiac failure, *DVT* deep venous thrombosis

All the study patients were evaluated for inherited thrombogenic risk factors as shown in Table 2.

**Table 2:** Etiological (inherited) profile of the study group

	Control group	Thrombolysis group	***p*** value
Factor V Leiden	2 (4.5)	2 (4.8)	0.962
Prothrombin gene mutation	0 (0)	0 (0)	–
Anti-thrombin III deficiency	0 (0)	0 (0)	–
Protein C deficiency	0 (0)	1 (2.4)	0.303
Protein S deficiency	0 (0)	3 (7.1)	0.071
ANA/APLA	4 (9.1)	3 (7.1)	0.741
Homocysteinemia	1 (2.3)	3 (7.1)	1.149
JAK2	0 (0)	2 (4.8)	0.143

*p* value < 0.05, statistically significant. *ANA* antinuclear antibody, *APLA* antiphospholipid antibody syndrome

Various ECG abnormalities and biomarkers of pulmonary embolism found in the study are given in Table 3.

**Table 3:** ECG abnormalities in the study and the control groups

		Control group	Thrombolysis group	***p*** value*
Sinus tachycardia		14 (31.8)	36 (85.7)	0.0001
S1Q3T3		5 (11.4)	3 (7.1)	0.501
RBBB		7 (15.9)	4 (9.5)	0.375
Incomplete RBBB		2 (4.5)	3 (7.1)	0.607
T wave inversion		8 (18.2)	8 (19)	0.918
Troponin T	Elevated	15 (34.1)	15 (35.7)	0.875
	Normal	29 (65.9)	27 (64.3)	
NT pro-BNP	Elevated	22 (50)	16 (38.1)	0.266
	Normal	22 (50)	26 (61.9)	

*p value < 0.05, statistically significant

Comparison of RV systolic dysfunction, RA/RV dilatation, and tricuspid regurgitation between the control and thrombolysis groups is shown in Table 4. The table shows echocardiographic parameters pre-treatment, post-treatment (thrombolysis plus heparin in the thrombolysis group vs. heparin alone in the control group), and at 30 days of follow-up. As shown in the table, there was a statistically significant improvement in RV dysfunction and normalization of RA/RV dilation in the thrombolysis group as compared to the control group.

**Table 4:** Comparison of RV functions in the control group vs. thrombolysis group

			Control group		Thrombolysis group	
			*N* (%)	*p* value	*N* (%)	*p* value
Dilated RA/RV	Pre vs. post		20 (45.5%) vs. 20 (45.5%) (*n* = 44)	1.000	26 (61.9%) vs. 11 (26.2%) (*n* = 42)	0.001*
	Pre vs. day 30		14 (40.0%) vs. 13 (37.1%) (*n* = 35)	1.000	21 (58.3%) vs. 6 (16.7%) (*n* = 36)	0.001*
	Post vs. day 30		14 (40.0%) vs. 13 (37.1%) (*n* = 35)	1.000	10 (27.8%) vs. 6 (16.7%) (*n* = 36)	0.344
RV systolic dysfunction	Pre vs. post		24 (54.5%) vs. 21 (47.7%) (*n* = 44)	0.629	22 (52.4%) vs. 8 (19.0%) (*n* = 42)	0.004*
	Pre vs. day 30		19 (37.1%) vs. 11 (31.4%) (*n* = 35)	0.096	19 (52.8%) vs. 3 (8.3%) (*n* = 36)	0.0001*
	Post vs. day 30		19 (54.3%) vs. 11 (31.4%) (*n* = 35)	0.057	5 (13.9%) vs. 3 (8.3%) (*n* = 36)	0.727
Tricuspid regurgitation	Pre	Mild	5 (11.4%)	0.064	7 (16.7%)	0.0001*
		Moderate	29 (65.9%)		22 (52.4%)	
		Sever	10 (22.7%)		13 (31.0%)	
	Post	Mild	15 (34.1%)		32 (76.2%)	
		Moderate	22 (50.0%)		10 (23.8%)	
		Sever	7 (15.9%)		0 (0.0%)	
	Pre	Mild	4 (11.4%)	0.011*	6 (16.7%)	0.0001*
		Moderate	22 (62.9%)		20 (55.6%)	
		Sever	9 (25.7%)		10 (27.8%)	
	Day 30	Mild	15 (42.9%)		23 (63.9%)	
		Moderate	15 (42.9%)		13 (36.1%)	
		Sever	5 (14.3%)		0 (0.0%)	
	Post	Mild	12 (34.3%)	0.678	26 (72.2%)	0.678
		Moderate	18 (51.4%)		10 (27.8%)	
		Sever	5 (14.3%)		0 (0.0%)	
	Day 30	Mild	15 (42.9%)		23 (63.9%)	
		Moderate	15 (42.9%)		13 (36.1%)	
		Sever	5 (14.3%)		0 (0.0%)	

*p value < 0.05, statistically significant

*Pre* pre-treatment, *post* post-treatment (within 48 h of treatment)

The comparative impact of thrombolysis vs. anticoagulation alone on the decrease in pulmonary artery systolic pressures (PASP) is given in Table 5. There was a statistically significant decrease both in the thrombolysis group and the control group between pre- and post-echocardiography. There was no statistically significant decrease in both the arms at day 30.

**Table 5:** Pulmonary artery pressures

	Control group		Thrombolysis group	
	PASP (mean ± SD)	***p*** value	PASP (mean ± SD)	***p*** value
Pre vs. post	64.4 ± 15.0 vs. 45.9 ± 9.9	0.0001*	68.3 ± 17.4 vs. 31.4 ± 6.9	0.0001*
Pre vs. day 30	64.5 ± 15.3 vs. 45.9 ± 11.5	0.0001*	68.2 ± 17.5 vs. 29.7 ± 6.4	0.0001*
Post vs. day 30	45.1 ± 10.8 vs. 45.9 ± 11.5	0.780	31.3 ± 7.3 vs. 29.7 ± 6.4	0.291

*p value < 0.05, statistically significant

Table 6 shows the impact of thrombolysis vs. anticoagulation alone on the primary outcomes of death, hemodynamic decompensation, and need for mechanical ventilation and on the incidence of ischemic stroke and recurrent PE.

**Table 6:** Outcome of the study population

	Control group	Thrombolysis group	Total	***p*** value
Death	5 (11.4)	1 (2.4)	6 (7.0)	0.102
Hemodynamic decompensation	6 (13.6)	1 (2.4)	7 (8.1)	0.046*
Mechanical ventilation	6 (13.6)	1 (2.4)	7 (8.1)	0.046*
Bleeding (day 07)	0 (0)	1 (2.4)	1 (1.2)	0.303
Ischemic stroke (days 0 and 7)	0 (0)	1 (2.4)	1 (1.2)	0.303
Recurrent PE	0 (0)	0 (0)	0 (0)	–

*p value < 0.05, statistically significant

The univariate and multivariate logistic regression analyses of the clinical conditions (CCF, COPD, active cancer, immobilization, estrogen use, and DVT) associated with the submassive pulmonary embolism showed that no parameter predicted mortality and was statistically significant. However, COPD and active cancer show odds ratio of more than 1.2.

## Discussion

This was a comparative observational study in which the impact of thrombolytic therapy combined with heparin vs. heparin alone in the treatment of acute submassive pulmonary embolism was evaluated. Nearly 2/3 of the patients in the study were males. The mean age of the patients in the study was 59.83 years. Similar demographic features were reported by Davidsingh et al. [16] in their study. Sinus tachycardia was the most common ECG finding noted. Classical S1Q3T3 ECG pattern was seen in less than 10% of patients. Significant elevation in markers of RV systolic dysfunction (NT pro-BNP) and RV myocardial injury (troponin T) was seen in both arms of the study with no significant difference with *p* value of 0.266 and 0.875, respectively. On careful evaluation, underlying risk factor for development of acute pulmonary embolism could be elicited in 96% of cases as demonstrated by multiple international studies [17]. In this study, the most common identified risk factor for PE was the presence of underlying CCF followed by COPD. APLA was identified as the most common thrombophilic risk factor followed by factor V Leiden mutation and homocysteinemia. Prothrombin gene mutation and anti-thrombin III mutation were not seen in any patient. Overall, the underlying risk factor could be identified in 67.44% of cases. Risk factor identification in this study is higher than other similar studies from India likely due to an extensive evaluation of patients in this study [17].

On the evaluation of the primary outcome of this study, mortality, hemodynamic decompensation (defined by the need for CPR, persistent SBP < 90 mmHg, or signs of shock) within 7 days of enrollment in the study, and need for mechanical ventilation, there was no statistically significant difference in the primary outcome of death in both the arms (*p* = 0.102). However, there were clinically significant numbers of deaths in the control group (5 deaths) compared with the thrombolysis group (1 death) out of total of 6 deaths. There was a significant difference (*p* = 0.046) in the number of patients who experienced hemodynamic decompensation with 1 patient in the thrombolysis group and 6 patients in the control group developing this condition. Similarly, a significant difference (*p* = 0.046) in the number of patients who needed mechanical ventilation was observed, with 1 patient in the thrombolysis group and 6 patients in the control group needing mechanical ventilation. The results of our study were in conformity with the results of MAPPET-3 Trial [18], which showed that there was no statistically significant difference in the primary outcome of death between the thrombolysis and the control groups. Similarly, there was a statistically significant decrease in the hemodynamic decompensation and need for mechanical ventilation in the thrombolysis group vs. the control group.

On the evaluation of secondary outcomes of this study, it was revealed that there was no statistically significant difference in the secondary outcomes like major bleeding, ischemic stroke, and recurrent PE within 7 days of hospital stay. This study showed a lower incidence of major bleed and stroke in comparison to PEITHO trial [19]. On comparison of thrombolysis plus heparin vs. heparin plus placebo, PEITHO trial showed major extracranial bleeding in 6.3% vs. 1.2% (*p* < .001) and stroke in 2.4% vs. 0.2% (*p* = .004) at 7 days. This may be related to using a higher dose of tenecteplase (vs. alteplase in this study) than needed in their study population. However, further studies are needed to confirm this.

There was a significant improvement (*p* = 0.001) in the degree of RA/RV dilatation in the thrombolysis group compared with the control group within the index hospital stay. And also, there was a significant (*p* = 0.001) improvement in the RA/RV dilatation in the thrombolysis group compared with the control group at day 30. Similar findings were noted by Kline et al. [20], Fasullo et al. [21], and Sinha et al. [22] in their studies. However, there was no significant improvement between post-treatment echo values and values at day 30. There was also a significant improvement (*p* = 0.004) in the RV systolic dysfunction [defined by tricuspid annular systolic velocity (TASV) < 10 cm/s] and in the severity of tricuspid regurgitation, in the thrombolysis group compared with the control within 7 days of hospital stay. However, there was no significant improvement between post-treatment echo values and values at day 30 in both the conditions, strengthening the fact that early intervention by thrombolysis translates into better RV outcomes.

In the current study, there was a significant reduction (*p* = 0.0001) in the pulmonary artery systolic pressure (PASP) in the thrombolysis group compared with the control group within 7 days of hospital stay. There was a significant reduction (*p* = 0.0001) in the PASP between the pre-thrombolysis echocardiographic value and value at day 30. In a similar study, the Moderate Pulmonary Embolism Treated with Thrombolysis (MOPETT) trial [13], a single center randomized trial involving 121 patients, pulmonary hypertension developed in 16% of the anticoagulation plus alteplase group vs. 57% of the anticoagulation-only group (*p* < .001). In our study, there was a reduction in PASP at 48 h in both the control and the thrombolysis groups but the significance persisted (*p* = 0.0001) only in the thrombolysis group at day 30. A similar conclusion was made by Kline et al. [17] and Jaff et al. [23] in their studies, concluding that early fall in elevated PAPs is critical to its credibility as a surrogate for PE-related mortality and much of the improvement in RV function possibly relates to more rapid relief of RV afterload due to better clot resolution, which in itself is associated with lower 6-month mortality.

## Limitation of the study

The main limitation of this study is that the study is a single-center study. A multicenter study with an even larger sample size may better validate the results.

## Conclusion

Thrombolysis in submassive pulmonary embolism is associated with an overall better outcome, with a significant improvement in the right ventricular function and a significant decrease in the pulmonary artery pressures with an insignificant effect on mortality and major bleed.

## Data Availability

All the data used in this study is available with the corresponding author.

## Abbreviations

DVT: Deep venous thrombosis
PE: Pulmonary embolism
CCF: Congestive cardiac failure
COPD: Chronic obstructive pulmonary disease
RA: Right atrium
RV: Right ventricle
PASP: Pulmonary artery systolic pressure
APLA: Antiphospholipid antibody
ANA: Antinuclear antibody
